# Diabetes does not affect outcome in patients with *Enterobacteriaceae *bacteremia

**DOI:** 10.1186/1471-2334-9-94

**Published:** 2009-06-13

**Authors:** Galo Peralta, M Blanca Sánchez, M Pía Roiz, J Carlos Garrido, Ramón Teira, Fátima Mateos

**Affiliations:** 1 Director del Instituto de Formación e Investigación Marqués de Valdecilla (IFIMAV), Avda de Valdecilla s/n, 39008 Santander, Spain; 2 Clinical Pharmacology Service, Hospital Universitario "Marqués de Valdecilla", Santander, Spain; 3 Microbiology Service, Hospital Sierrallana, Torrelavega, Spain; 4 Biochemistry Service, Hospital Sierrallana, Torrelavega, Spain; 5 Internal Medicine Service, Hospital Sierrallana, Torrelavega, Spain

## Abstract

**Background:**

There is limited information about the effect of diabetes on the prognosis of patients with bacterial infections. We performed a retrospective cohort study to investigate possible correlations between diabetes and prognosis in patients with *Enterobacteriaceae *bacteremia.

**Methods:**

We reviewed the medical charts of 1112 patients who were treated at a community teaching hospital for *Enterobacteriaceae *bacteremia from January 1997 through June 2007. Factors associated with in-hospital mortality were analyzed by logistic regression analysis.

**Results:**

Among the 1112 patients with *Enterobacteriaceae *bacteremia, 181 (16.3%) were diabetic patients; 90 patients (8.1%) died while in the hospital. Compared to non-diabetic patients, diabetic patients were older (75.4 ± 11.9 years vs. 70 ± 16.6 years, *p *< 0.001) and had more comorbidities. However, mortality among diabetic and non-diabetic patients was not different [7.2% vs. 8.2%, RR 1.13; 95% CI (0.67–1.9); *p *= 0.39]. In a multivariate analysis, the variables associated with in-hospital mortality were age, the origin of the bacteremia, and the presence of immunosuppression. Diabetes was not associated with outcome.

**Conclusion:**

In this cohort of patients with *Enterobacteriaceae *bacteremia, diabetes was not associated with a poorer prognosis.

## Background

*Enterobacteriaceae *are the dominant causal agents of Gram-negative bacteremia and are a major cause of morbidity and mortality [1]. The prevalence of diabetes is increasing worldwide [2]; considering the predisposition of diabetes patients to *Enterobacteriaceae *bacteremia [3], the number of patients with both diabetes and bacteremia caused by *Enterobacteriaceae *is likely to increase as well. However, information about the effect of diabetes on the prognosis of patients with infections in general, and with *Enterobacteriaceae *bacteremia in particular, is limited. In addition, some data are contradictory [3-6]. It is significant to determine whether diabetes has a negative impact on the prognosis of *Enterobacteriaceae *bacteremia so this would support the convenience of a more aggressive approach for these patients. We performed a retrospective cohort study to identify possible correlations between diabetes and prognosis in patients with *Enterobacteriaceae *bacteremia.

## Methods

The Sierrallana Hospital is an adult acute-care center in Torrelavega, a city in the province of Cantabria, Spain that forms part of a health district of 160,000 inhabitants. It is a teaching institution with 250 beds that has approximately 8000 admissions and 65000 assistances at the emergency service annually. It includes most major departments and specialties, except transplantation, thoracic, cardiovascular surgery and neurosurgery units. Using the microbiology laboratory database, we identified patients with *Enterobacteriaceae *bacteremia who were treated at Hospital Sierrallana, from January 1997 through June 2007. Only the first episode of bacteremia in a particular patient, accounted at our hospital during the period of study, was included.* Enterobacteriaceae *strains recovered from blood samples were identified and tested for antimicrobial susceptibility by the automated testing systems MicroScan (Dade Behring, Sacramento, CA, USA) or BD Phoenix (Becton-Dickinson Biosciences, Sparks, MD, USA).

A standardized data collection form was used to review the hospital records of the included patients. Diabetes was considered present if there was a previous diagnosis of type 1 or type 2 diabetes, or if diabetes was diagnosed during the patient's treatment for bacteremia. For the diagnosis of diabetes the American Diabetes Association diagnostic criteria were used [7]. Renal insufficiency was indicated by a creatinine value ≥ 2.0 mg/dL. Neutropenia was defined as an absolute neutrophil count of <500 cells/mm^3 ^at the bacteremia onset. Immunosuppression was defined as the presence of neutropenia, infection with the human immunodeficiency virus or current treatment with immunosuppressive agents. Sepsis, severe sepsis and septic shock were defined according to the guidelines of Bone et al [8]. The initial empirical antimicrobial therapy was considered appropriate if the antibiotics were administered within 24 h of acquisition of a blood culture sample and included at least one antibiotic that was active in vitro against the causative microorganism(s). Therapy with urinary antiseptics such as norfloxacin, fosfomycin, pipemidic acid or nalidixic acid was considered inadequate.

A significance level of 0.05 (2-sided) was used for all tests. Relative risk (RR) was calculated as the ratio of the incidence rate in patients exposed to a risk factor to the incidence rate in those not exposed. Univariate analyses and logistic regression were performed to identify factors associated with patient death. Factors were added to the model using variables with a *p-*value <0.1 in the univariate analysis. Diabetes was included as a variable in the regression model independent of the *p*-value. The statistical analyses were performed using SPSS software, version 14.0. The study was approved by the Comité Etico de Investigación Clínica de Cantabria.

## Results

During the study period, 1134 cases of *Enterobacteriaceae *bacteremia were identified; of these, full medical records were available for 1112 (98%). We identified 181 (16.3%) patients with a diagnosis of diabetes, and 90 (8.1%) patients died while undergoing treatment for bacteremia. The following *Enterobacteriaceae *were isolated from the blood cultures: *Escherichia coli *in 862 (77.5%) patients, *Klebsiella *spp. in 82 (7.4%) patients, *Proteus mirabilis *in 51 (4.6%) patients, *Salmonella *spp. in 42 (3.8%) patients, *Enterobacter *spp. in 36 (3.2%) patients, *Morganella morgagnii *in 12 (1.1%) patients, *Citrobacter *spp. in 11 (1%) patients, *Serratia marcescens in 9 (0.8%) *patients, *Pantoea agglomerans *in 5 (0.4%) patients and *Leclercia adecarboxylata and Yersinia enterocolitica *in one patient each. Comparing the characteristics of diabetic and non-diabetic patients, significant differences in sex, age, comorbidities and initial blood glucose concentration were detected (Table 1). The proportion of patients with bacteremia caused by *Klebsiella *spp. reached borderline statistical significance. Mortality was 7.2% (13/181) for diabetic patients and 8.2% (76/927) for non-diabetic patients (*p *= 0.39).

**Table 1:** Characteristics of diabetic and non-diabetic patients with bacteremia due to *Enterobacteriaceae*.

	Diabetics n = 181	Non-diabetics n = 931	*p*-value	RR* (95% CI)
**Demographics**				

Male	88 (48.6)	521 (51.4)	0.07	0.78 (0.59–1.01)

Age, years (mean ± SD)	75.4 ± 11.9	70 ± 16.6	<0.001	-

Nosocomial	24 (13.3)	119 (13)	0.9	1.02 (0.69–1.51)

ICU-acquired	1 (0.6)	10 (1.1)	1	0.55 (0.09–3.6)

Treated in ICU	8 (4.4)	48 (5.2)	0.42	0.87(0.45–1.67)

Postsurgical	4 (2.2)	33 (3.6)	0.5	0.65 (0.26–1.67)

**Severe sepsis or shock**	32 (17.7)	130 (14)	0.21	1.25 (0.89–1.77)

**Adequate empirical antibiotic**	150 (84.7)	751 (81.6)	0.39	1.21 (0.83–1.77)

**Blood glucose concentration**, mg/dL (mean ± SD)	236 ± 103	143 ± 50	<0.001	-

**Comorbidities**				

COPD**	25 (13.8)	89 (9.6)	0.12	1.4 (0.96–2.03)

Ictus	15 (8.3)	48 (5.2)	0.11	1.5 (0.94–2.38)

Cirrhosis	11 (6.1)	50 (5.4)	0.72	1.11 (0.64–1.93)

Chronic renal failure	12 (6.6)	24 (2.6)	0.01	2.11 (1.31–3.43)

Immunosuppression	4 (2.2)	48 (5.2)	0.12	0.46 (0.18–1.19)

Cardiac failure	19 (10.5)	41 (4.4)	0.002	2.05 (1.38–3.05)

**Origin of infection**				

Urinary	100 (55.9)	479 (51.9)	0.37	1.14 (0.87–1.5)

Biliary	34 (19)	166 (18)	0.75	1.06 (0.75–1.49)

Unknown	24(13.4)	138 (15)	0.65	0.9 (0.6–1.34)

Intraabdominal	7 (3.9)	52 (5.6)	0.47	0.72 (0.35–1.46)

Pneumonia	4 (2.2)	26 (2.8)	0.81	0.82 (0.33–2.05)

**Microorganism**				

*E. coli*	135 (74.6)	726 (78.3)	0.28	0.84 (0.62–1.14)

*Klebsiella *spp.	19 (10.5)	62 (6.7)	0.09	1.49 (0.98–2.26)

Other	27 (14.9)	139 (15)	1	1 (0.69–1.461

Polymicrobial	20 (11)	70 (7.6)	0.14	1.41 (0.93–2.12)

**Death**	13 (7.2)	76 (8.2)	0.77	1.13 (0.67–1.9)

**Length of hospital stay**, days (mean ± SD)	13.3 ± 12	13.9 ± 15	0.64	

Data are n (%) otherwise indicated

*RR: Relative risk

**COPD: chronic obstructive pulmonary disease

Variables significantly associated with mortality in the univariate analysis are shown in Table 2. As nosocomial and community acquired bacteremia are quite different entities in terms of risk factors and prognosis a stratified analysis was performed analyzing variables associated with prognosis in these subgroups (Table 3 and Table 4).

**Table 2:** Risk factors for mortality in the entire cohort.

	Exitus n = 90	Surviving n = 1022	*p*-value	RR* (95% CI)
**Demographics**				

Male	55 (61.1)	558 (54.6)	0.14	1.27 (0.85–1.91)

Age, years (mean ± SD)	76.8 ± 12.47	70.4 ± 16.2	<0.001	

**Severe sepsis or shock**	52 (58.4)	110 (10.8)	<0.001	8.21 (5.57–12.1)

**Adequate empirical antibiotic**	63 (70.8)	838 (83)	0.004	0.53 (0.34–0.81)

**Blood glucose concentration**, mg/dL (mean ± SD)	164 ± 78	157 ± 70	0.85	

**Comorbidities**				

COPD**	10 (11.2)	104 (10.2)	0.44	1.1 (0.59–2.07)

Ictus	8 (9)	55 (5.4)	0.13	1.64 (0.83–3.24)

Cirrhosis	7 (7.9)	54 (5.3)	0.21	1.47 (0.71–3.03)

Chronic renal failure	9 (10)	28 (2.7)	0.006	2.94 (1.54–5.61)

Immunosuppression	10 (11.2)	42 (4.1)	0.006	2.57 (1.42–4.67)

Cardiac failure	11 (12.4)	49 (4.8)	0.006	2.46 (1.39–4.38)

Diabetes	13 (14.6)	168 (16.5)	0.39	0.88 (0.5–1.54)

**Origin of infection**				

Urinary	19 (21.6)	561 (55.1)	<0.001	0.25 (0.15–0.41)

Biliary	16 (18.2)	185 (18.2)	0.55	1 (0.6–1.68)

Unknown	22 (25)	140 (13.8)	0.007	1.89 (1.20–2.97)

Intraabdominal	7 (8)	52 (5.1)	0.18	1.89 (0.74–3.17)

Pneumonia	14 (15.9)	16 (1.6)	<0.001	6.79 (4.37–10.6)

**Microorganism**				

*E. coli*	57 (63.3)	805 (78.8)	0.001	0.5 (0.33–0.75)

*Klebsiella *spp.	9 (10)	73 (7.1)	0.21	1.4 (0.73–2.68)

*Proteus *spp.	5 (5.6)	47 (4.6)	0.41	1.2 (0.51–2.83)

*Citrobacter *spp.	2 (2.2)	9 (0.9)	0.22	2.28 (0.64–8.1)

*Enterobacter *spp.	6 (6.7)	30 (2.9)	0.06	2.14 (1–4.56)

*Serratia marcescens*	2 (2.2)	7 (0.7)	0.16	2.79 (0.81–9.61)

*Morganella morgagnii*	3 (3.3)	9 (0.9)	0.07	3.16 (1.16–8.6)

*Salmonella *spp.	5 (5.6)	37 (3.6)	0.25	1.5 (0.64–3.5)

Polymicrobial	14 (15.6)	76 (7.4)	0.01	2.09 (1.23–3.54)

Univariate risk estimates are given

Data are n (%) otherwise indicated

*RR: Relative risk.

**COPD: chronic obstructive pulmonary disease

**Table 3:** Risk factors for mortality in patients with nosocomial bacteremia

	Exitus n = 19	Surviving n = 124	*p*-value	RR* (95% CI)
**Demographics**				

Male	15 (78.9)	76 (61.3)	0.03	2.14 (0.75–6.12)

Age, years (mean ± SD)	73 ± 9.27	71.9 ± 13.75	0.01	

**Severe sepsis or shock**	11 (57.9)	19 (15.2)	<0.001	5.18 (2.31–11.8)

**Adequate empirical antibiotic**	16 (80)	93 (78.8)	0.59	1.37 (0.43–4.38)

**Blood glucose concentration**, mg/dL (mean ± SD)	138.2 ± 58.1	144.8 ± 64.8	0.95	

**Comorbidities**				

COPD**	5 (26.3)	17 (13.7)	0.17	1.96 (0.75–4.71)

Ictus	2 (10.5)	6 (4.8)	0.29	1.99 (0.56–7.19)

Cirrhosis	0	8 (6.4)	0.6	

Chronic renal failure	5 (26.3)	1 (0.8)	<0.001	8.16 (4.42–15.04)

Immunosuppression	2 (10.5)	5 (4)	0.23	2.29 (0.65–8)

Cardiac failure	5 (26.3)	12 (9.7)	0.05	2.65 (1.09–6.43)

Diabetes	3 (15.8)	21 (16.9)	0.6	0.93 (0.29–2.94)

**Origin of infection**				

Urinary	1 (5)	50 (40.3)	0.002	0.1 (0.01–0.72)

Biliary	2 (10.5)	22 (17.9)	0.34	1.07 (0.93–1.23)

Unknown	6 (30)	24 (20)	0.24	1.72 (0.72–4.15)

Intraabdominal	2 (10.5)	18 (14.6)	1	0.72 (0.18–2.87)

Pneumonia	5 (26.3)	3 (2.4)	0.001	5.67 (2.76–11.62)

**Microorganism**				

*E. coli*	8 (42.1)	89 (71.2)	0.01	0.34 (0.15–0.8)

*Klebsiella *spp.	4 (20)	19 (15.2)	0.51	1.39 (0.51–3.82)

*Proteus *spp.	1 (5.3)	3 (2.4)	0.44	1.93 (0.34–11.12)

*Citrobacter *spp.	1 (0.8)	0	0.86	

*Enterobacter *spp.	3 (15.8)	9 (7.3)	0.2	2.05 (0.69–6.04)

*Serratia marcescens*	0	3 (2.4)	1	

*Morganella morgagnii*	2 (10.5)	1 (0.8)	0.05	5.49 (2.19–13.71)

*Salmonella *spp.	0	0		

Polymicrobial	3 (15)	7 (5.6)	0.13	2.49 (0.87–7.14)

Univariate risk estimates are given

Data are n (%) otherwise indicated

*RR: Relative risk

**COPD: chronic obstructive pulmonary disease

**Table 4:** Risk factors for mortality in patients with community acquired bacteremia.

	Exitus n = 70	Surviving n = 894	*p*-value	RR (95% CI)
**Demographics**				

Male	39 (55.7)	479 (53.7)	0.8	1.08 (0.69–1.7)

Age, years (mean ± SD)	78.1 ± 12.8	70.1 ± 16.5	<0.001	

**Severe sepsis or shock**	41 (58.6)	91 (10.2)	<0.001	8.91 (5.75–13.82)

**Adequate empirical antibiotic**	47 (68.1)	746 (83.6)	0.002	0.45 (0.28–0.73)

**Blood glucose concentration**, mg/dL (mean ± SD)	170.9 ± 80.4	158.6 ± 70.2	0.44	

**Comorbidities**				

COPD**	5 (7.1)	86 (9.6)	0.67	0.74 (0.31–1.78)

Ictus	6 (8.6)	49 (5.5)	0.28	1.55 (0.7–3.42)

Cirrhosis	7 (10)	46 (5.1)	0.08	1.91 (0.84–1.04)

Chronic renal failure	3 (4.3)	27 (3)	0.47	1.39 (0.47–4.18)

Immunosuppression	8 (11.4)	37 (4.1)	0.01	2.64 (1.35–5.16)

Cardiac failure	6 (8.6)	36 (4)	0.08	2.06 (0.95–4.48)

Diabetes	10 (14.3)	146 (16.3)	0.4	0.86 (0.45–1.65)

**Origin of infection**				

Urinary	18 (26.5)	511 (57.1)	<0.001	0.3 (0.18–0.5)

Biliary	13 (19.1)	163 (18.2)	0.87	1.06 (0.59–1.89)

Unknown	16 (23.5)	120 (13.4)	0.03	1.87 (1.1–3.18)

Intraabdominal	5 (7.4)	33 (3.7)	0.18	1.93 (0.83–4.52)

Pneumonia	9 (13.2)	13 (1.5)	<0.001	6.53 (3.73–11.42)

**Microorganism**				

*E. coli*	48 (68.6)	716 (79.8)	0.03	0.58 (0.36–0.94)

*Klebsiella *spp.	5 (7.1)	54 (6)	0.43	1.18 (0.5–2.83)

*Proteus *spp.	4 (5.7)	44 (4.9)	0.46	1.16 (0.44–3.05)

*Citrobacter *spp.	2 (2.9)	8 (0.9)	0.16	2.82 (0.8–9.93)

*Enterobacter *spp.	3 (4.3)	21 (2.3)	0.25	1.76 (0.6–5.2)

*Serratia marcescens*	2 (2.9)	4 (0.4)	0.06	4.71 (1.49–14.95)

*Morganella morgagnii*	1 (1.4)	8 (0.9)	0.49	1.54 (0.24–9.93)

*Salmonella *spp.	5 (7.1)	37 (4.1)	0.18	1.69 (0.72–3.99)

Polymicrobial	11 (15.7)	68 (7.6)	0.02	2.09 (1.15–3.82)

Data are n (%) otherwise indicated

RR: Relative risk

*COPD: chronic obstructive pulmonary disease

Variables that were associated with in-hospital mortality in the multivariate analysis in the whole cohort, in nosocomial cases and in community acquired cases are reflected in Table 5. The severity of sepsis was not included in the initial models [9]. Diabetes was not associated with outcome in these analyses. In a second analysis including the severity of sepsis in the whole cohort, in the nosocomial cases and in community acquired cases, diabetes was not associated with outcome.

**Table 5:** Results of the multivariate analysis for in-hospital mortality in the overall cohort, in community acquired cases and in nosocomial cases.

	ORa (95% CI)	*p*-value
**Overall**		

Age	1.04 (1.02–1.06)*	<0.001

Urinary origin	0.031 (0.17–0.54)	<0.001

Immunosuppression	3.23 (1.34–7.81)	0.009

Diabetes	0.64 (0.3–1.3)	0.21

**Community acquired cases**		

Age	1.06 (1.03–1.09)	<0.001

Urinary origin	0.32 (0.18–0.59)	<0.001

Immunosuppression	3.26 (1.2–8.81)	0.02

Adequate empirical antibiotic	0.51 (0.26–0.98)	0.04

Cirrhosis	4.61 (1.65–12.87)	0.004

Diabetes	0.62 (0.28–1.38)	0.24

**Nosocomial cases**		

Urinary origin	0.09 (0.01–0.94)	0.04

Chronic renal failure	33.8 (1.3–883)	0.03

Diabetes	0.45 (0.05–4.02)	0.47

Table reflects only variables associated significantly with in-hospital mortality and diabetes.

The variable "severe sepsis or shock" was not included in the model.

aOR: adjusted odds ratio

*Per increment of one year

## Discussion

Diabetes has traditionally been associated with a worse prognosis for patients with infectious diseases such as *Enterobacteriaceae *bacteremia [3], liver abscess [4] and tuberculosis [6]. However, the prognosis of diabetic patients has improved in recent years for some procedures, such as coronary bypass grafting [10], and for some conditions, such as myocardial infarction [11]. This improvement has been attributed, at least in part, to better glucose control in diabetics. A similar improvement would be expected for the prognosis of diabetic patients with bacterial infections, which is what we found in this study. Another recent study also found no differences in the outcomes of diabetic patients with community-acquired bacteremia [5]. Improvements in glucose control in diabetic patients may account for the discordance between our results and those of Thomsen et al [3]; this group found a slightly higher risk of late death in diabetic patients in a population study of patients with *Enterobacteriaceae *bacteremia conducted from 1992 through 2001. Other explanations could be related to differences in the population analyzed, comorbidities and the adequacy of antibiotic empirical treatment. We included some variables that were not analyzed by Thomsen et al, which could also contribute to the difference in results. In addition, we only evaluated the effect of diabetes on in-hospital mortality, not on 30- and 90-day mortality [3]. Our study is the first one which analyzes the mortality of diabetic patients with bacteremia performed in Mediterranean country. The protective effect of the Mediterranean diet over our diabetic patients with bacteremia cannot be discarded.

The mortality in our series is lower that previous similar studies [3]. Several factors, such as a lower incidence of co-morbidity, the low proportion of patients with septic shock and the high proportion of adequate empirical treatment among our patients with *Enterobacteriaceae *bacteraemia may contribute to the low mortality [12]. The incidence of Enterobacteriaceae bacteremia in our series in much higher than in others [3,13]; this suggest that the lower mortality are due to a more vigilant attitude in our centre that lead to the detection of more mild cases of bacteremia.

Our study has several potential limitations. The differences in mortality could be lower that the sample size would permit to detect. As the data, including the diagnosis of diabetes has been obtained retrospectively, this could lead to information bias between exposure and outcome, so the diagnosis of diabetes may actually have been missed in patients dying precipitously, and conversely there is a risk that a diagnosis of diabetes has been wrongly inferred from hyperglycemia in some surviving patients. We did not routinely collect measures of long-term glucose control, such as HbA1c, which would have allowed us to distinguish the effects of long-term poorly-controlled diabetes on patient prognosis. Finally, our population was mainly elderly people with type II diabetes, thus, these results may not be applicable to patients diagnosed with type I diabetes.

Our data suggest that the presence of diabetes is not related to in-hospital mortality in patients with *Enterobacteriaceae *bacteremia. Improved management of diabetic patients with acute illnesses in recent years may account for the absence of an effect of diabetes on outcomes in these patients.

## Conclusion

In conclusion, in our cohort of patients with *Enterobacteriaceae *bacteremia, diabetes was not associated with a poorer prognosis. Improved management of diabetic patients with acute illnesses in recent years may account for the absence of an effect of diabetes on outcomes in these patients.

## Competing interests

Galo Peralta has been a consultant for Janssen-Cilag, Wyeth, Bristol-Myers Squibb, and Boehringer Ingelheim, and has also served as a speaker for Wyeth and GlaxoSmithKline.

## Authors' contributions

GP, MPR, MBS, and JCG were involved in the study conception, RT and FT were involved in the coordination, and data acquisition, GP performed the data analyses, all authors were involved in the interpretation and validation of the results, GP, MPR and MBS were involved in the drafting of the manuscript and all authors read and approved the final manuscript.

## Pre-publication history

The pre-publication history for this paper can be accessed here:

http://www.biomedcentral.com/1471-2334/9/94/prepub
